# Forging property, processing map, and mesoscale microstructural evolution modeling of a Ti-17 alloy with a lamellar (α+β) starting microstructure

**DOI:** 10.1080/14686996.2017.1386530

**Published:** 2017-11-03

**Authors:** Hiroaki Matsumoto, Daiki Naito, Kento Miyoshi, Kenta Yamanaka, Akihiko Chiba, Yoko Yamabe-Mitarai

**Affiliations:** ^a^ Department of Advanced Materials Science, Faculty of Engineering, Kagawa University, Takamatsu, Japan; ^b^ Institute for Materials Research, Tohoku University, Sendai, Japan; ^c^ Research Center for Structural Materials, National Institute for Materials Science, Tsukuba, Japan

**Keywords:** Ti-5Al-2Sn-2Zr-4Mo-4Cr alloy, dynamic globularization, microstructural prediction, processing map, finite element simulation, 10 Engineering and Structural materials, 106 Metallic materials, 303 Mechanical / Physical processing, 402 Multi-scale modeling, 503 TEM, STEM, SEM

## Abstract

This work identifies microstructural conversion mechanisms during hot deformation (at temperatures ranging from 750 °C to 1050 °C and strain rates ranging from 10^−3^ s^−1^ to 1 s^−1^) of a Ti-5Al-2Sn-2Zr-4Mo-4Cr (Ti-17) alloy with a lamellar starting microstructure and establishes constitutive formulae for predicting the microstructural evolution using finite-element analysis. In the α phase, lamellae kinking is the dominant mode in the higher strain rate region and dynamic globularization frequently occurs at higher temperatures. In the *β* phase, continuous dynamic recrystallization is the dominant mode below the transition temperature, *T*_*β*_ (880~890 °C). Dynamic recovery tends to be more active at conditions of lower strain rates and higher temperatures. At temperatures above *T*_*β*_, continuous dynamic recrystallization of the *β* phase frequently occurs, especially in the lower strain rate region. A set of constitutive equations modeling the microstructural evolution and processing map characteristic are established by optimizing the experimental data and were later implemented in the DEFORM-3D software package. There is a satisfactory agreement between the experimental and simulated results, indicating that the established series of constitutive models can be used to reliably predict the properties of a Ti-17 alloy after forging in the (α+*β*) region.

## Graphical abstract

**Figure:**
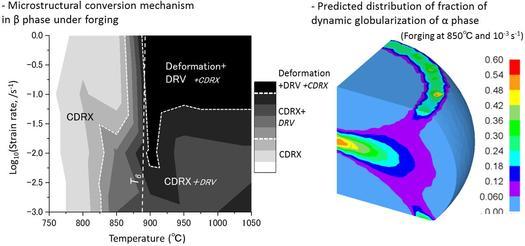

## 1. Introduction

Ti-5Al-2Sn-2Zr-4Mo-4Cr (Ti-17) alloy, a near-*β*-type (*α*+*β*) alloy, was first developed by GE Aviation [1]. Since then, the Ti-17 alloy has been widely used to manufacture fan blades and compressor disks in aircraft engines owing to its high strength, superior fracture toughness, and excellent creep property. During the hot deformation of near *α* and (*α*+*β*) Ti alloys, dynamic recovery (DRV) or continuous dynamic recrystallization (CDRX) is reported to be dominant [2]. Unlike discontinuous dynamic recrystallization (DDRX), CDRX occurs in a manner in which subgrains with low-angle boundaries are formed; these subgrains subsequently evolve into grains with a large fraction of high angle boundaries upon increasing the plastic strain.

During the hot deformation (low temperatures ranging from 750 °C to 900 °C) of a Ti-6Al-4 V alloy having a lamellar (*α*+*β*) starting microstructure, deformation proceeds with dynamic globularization, prior *β*-boundary cracking, lamellae kinking, and adiabatic shear banding/flow localization [2,3]. As in the case of the Ti-6Al-4 V alloy, dynamic globularization is the dominant mode for microstructural conversion during the hot deformation of the Ti-17 alloy (with a lamellar (*α*+*β*) starting microstructure) in the temperature range below the transition temperature, *T*_*β*_ (880~890 °C) [4–7]. Wang et al. [7] reported the prediction of the dynamic globularization behavior through a back-propagation artificial neural network (ANN) method. The nucleation sites for globularization are at the kinks in the lamellae as well as at some of the prior *β* grain boundaries. This type of microstructural change involving a reduction in the aspect ratio of the plates is interpreted in terms of geometric dynamic recrystallization, as proposed by McQueen et al. [8,9]. There is a large body of work on the numerical modeling of microstructural evolution during the hot deformation of Ti alloys [10–17]. In one of the studies, Sun et al. worked on the microstructural prediction of a TA15 alloy on the basis of the advanced dislocation-density-rate equation [12]. Additionally, the classical Johnson-Mehl-Avrami-Kolmogorov (JMAK) formulation enables us to predict the DRX behavior of Ti alloys [15–17].

In order to characterize the hot forging property of metals, construction of processing maps according to the dynamic materials model [18,19] is the generally followed approach. A processing map enables us to optimize the processing window for the hot forging process. Taking this need into account, several studies have been conducted on the processing map characteristic of the Ti-17 alloy [4,20].

The present work focuses on the microstructural predictions (dynamic globularization behavior and change in grain size) and modeling of a processing map characteristic for the forging of a Ti-17 alloy with a lamellar (*α*+*β*) starting microstructure. Initially, the microstructural conversion mechanism and the processing map characteristic for the hot forging of the Ti-17 alloy with a lamellar (*α*+*β*) starting microstructure were examined through detailed microstructural observations and analysis of deformation kinetics. Later, constitutive formulae were established on the basis of the experimental results and implemented in the finite-element method (FEM) software (DEFORM-3D, v.10.2).

## 2. Experimental procedures

### 2.1. Initial microstructure of the Ti-17 alloy and testing conditions of isothermal forging

**Figure 1.**
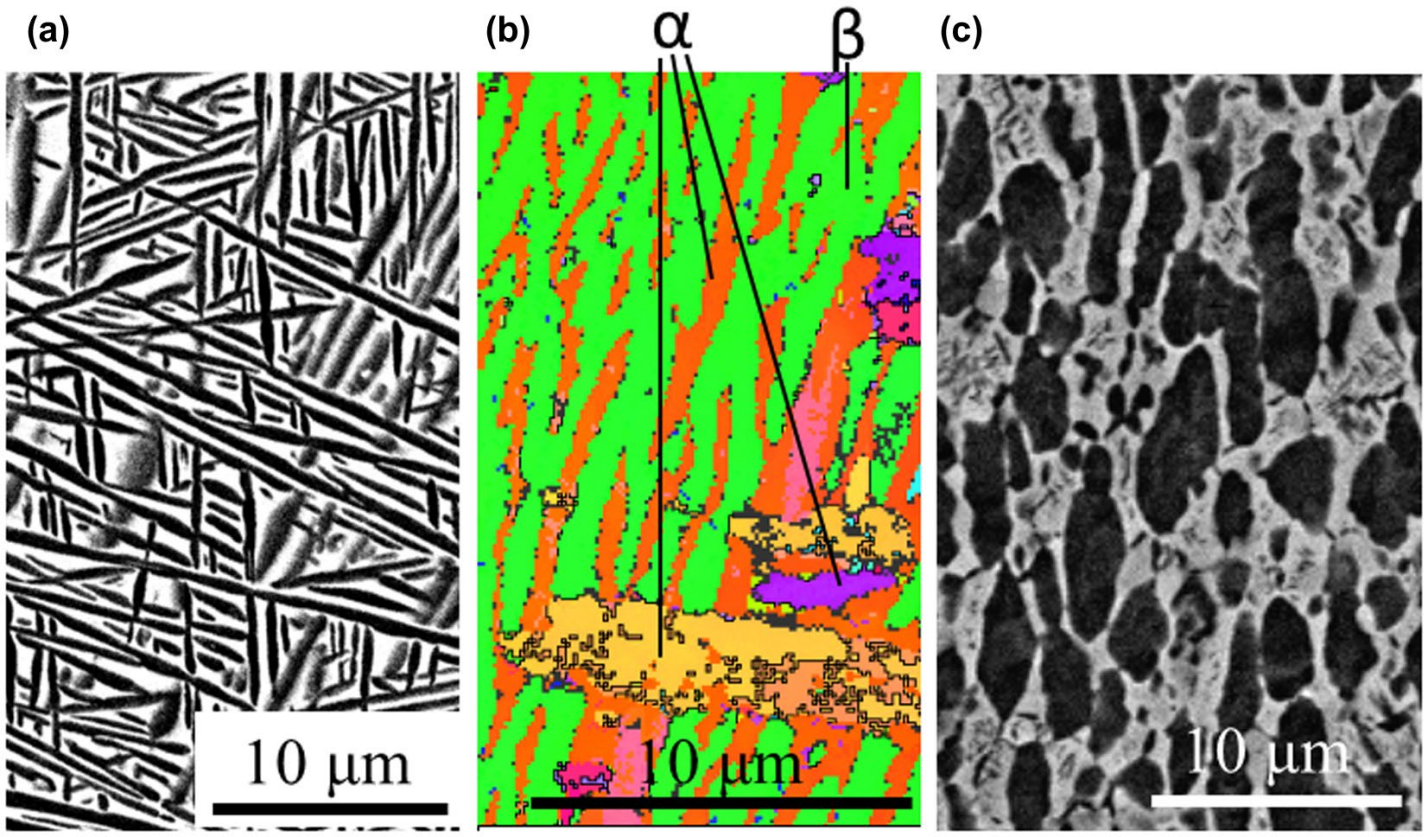
Starting microstructures of (a), (b) lamellar (*α*+*β*) microstructure and (c) equiaxed (*α*+*β*) microstructure of the Ti-17 alloy ((a) and (c) depict the SEM-BSE images and (b) depicts the EBSD-orientation image). In the SEM images, the white regions correspond to the *β* phase and the black regions correspond to the *α* phase.

A Ti-17 alloy with a chemical composition (wt.%) of Ti-4.89Al-2.00Sn-1.92Zr-3.82Cr-3.93Mo-(0.09Fe-0.014Si-0.102O-0.01C) was used in this work. Two types of starting microstructures, a lamellar (*α*+*β*) microstructure (as shown in Figure 1(a) and (b)) and an equiaxed (*α*+*β*) microstructure (as shown in Figure 1(c)) with an average *α*-grain size of 3 μm were used in this work. This study especially focuses on the forging behavior of the lamellar (*α*+*β*) starting microstructure. The microstructural analysis of Figure 1(a) and (b) revealed that the grain size of the coarse prior *β* grain was approximately 1 mm, the lamellar width of the *α* phase was approximately 0.46 μm, and that the average aspect ratio of the *α*-lamellae was 24. By image analysis, it was determined that the *β* phase fraction was 42% in the lamellar starting microstructure and 40% in the equiaxed starting microstructure.

An isothermal forging test was performed using an AUTOGRAPH AG-X plus precision universal tester (SHIMADZU) with a 50 kN load cell. During the forging test, the process temperatures were maintained at 750 °C, 800 °C, 850 °C, 900 °C, 950 °C and 1050 °C, while the strain rates ranged from 10^−3^ s^−1^ to 1 s^−1^. The specimens (5 mm diameter and 7.5 mm height) used for the forging test were fabricated by electron discharge machining. The specimens were heated at approximately 0.3 °C s^-1^ up to the testing temperature and maintained there for 10 min to ensure a uniform temperature distribution throughout the specimen. The forging test was conducted at a constant strain rate and a height true strain of approximately 0.75. After forging testing, the specimens were cooled in air for 10 s (to avoid a martensitic transformation) and then quenched in oil. The experimentally obtained stress–strain curves were corrected by the friction correction (based on the reports by Li et al. [21,22]) and adiabatic correction methods (based on the report by Mataya and Sackschewsky [23]) so as to obtain accurate stress–strain curves.

### 2.2. Microstructural observations

The microstructure was analyzed with a field emission scanning electron microscope (FE-SEM) fitted with an electron back-scattering diffraction (EBSD) analyzer equipped with the HKL Channel 5 software. From the EBSD results, the crystallographic orientation and fraction of low angle boundaries were evaluated. The OLYMPUS-Stream software was used on the SEM-backscattered electron image (BSE) or the EBSD image to evaluate the grain sizes of the *α*- and *β*-phases and the average aspect ratio of the *α* lamellae. For analyzing dynamic globularization during deformation, globularization was deemed to have occurred when the *α* lamellae exhibited an aspect ratio less than 4 (the initial average aspect ratio was 24). The microstructure was evaluated at five locations (as shown in Figure 10(a) later) on the forged specimen. The effective strain rate, strain and temperature at these locations were estimated by FEM using the DEFORM-3D software. The obtained microstructural data and FEM data were incorporated into an optimization process for determining the material constants in the constitutive equations, as described in Section 3.2 later.

### 2.3. Prediction of microstructures and processing map characteristic combined with FEM analysis

The forging characteristics of the Ti-17 alloy with a lamellar (*α*+*β*) starting microstructure were analyzed by FEM with a DEFORM-3D software (v. 10.2) using a user-defined subroutine. The simulated condition is basically similar to the experimental forging condition. The friction coefficient between the die-tools and the specimen was set to 0.7. Due to the symmetry of the cylindrical specimen (5 mm in diameter and 7.5 mm in height), a quarter of it was taken as the object (herein, the number of elements is 20,000) and the constraint was applied in the plane of symmetry. The corrected stress–strain data obtained in this work was programed into the FEM code. The constitutive equations for modeling the microstructure and processing map characteristic are summarized in Section 3.2.

## 3. Results and discussion

### 3.1. Experimental forging properties of the Ti-17 alloy with a lamellar (*α*+*β*) starting microstructure

#### 3.1.1. Flow behavior

**Figure 2.**
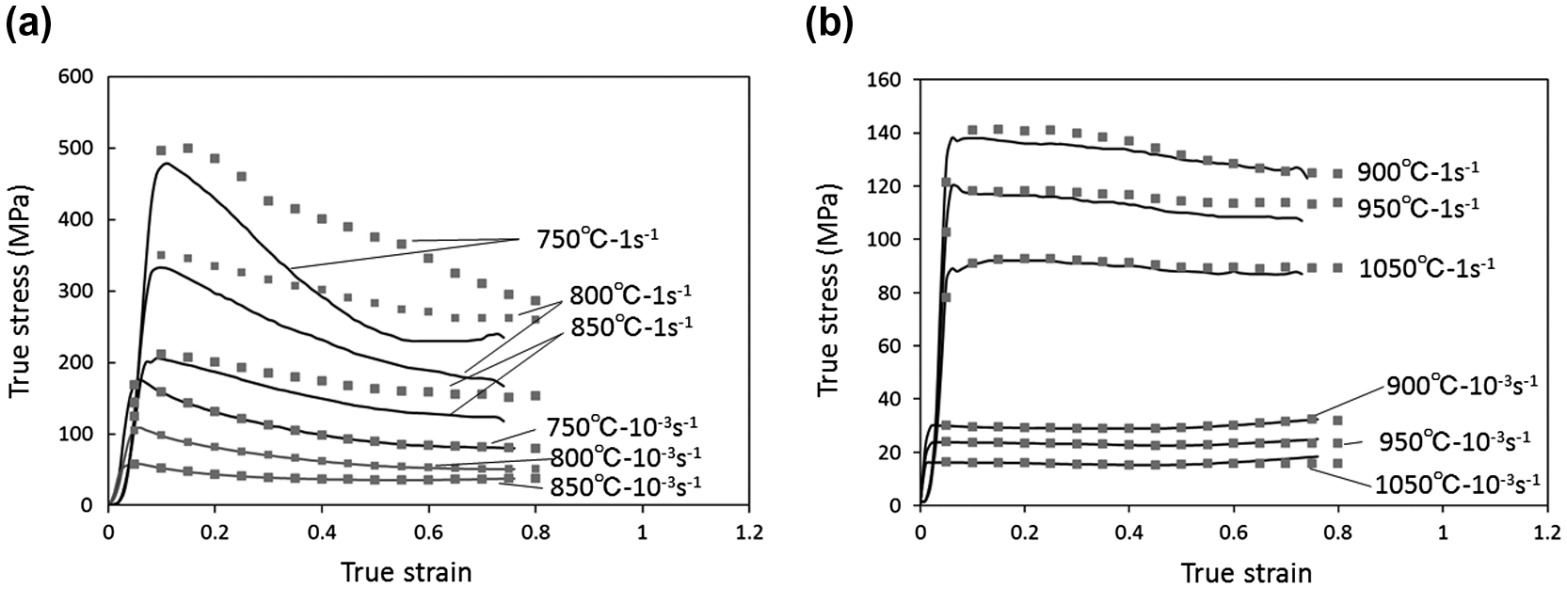
True stress–true strain curves (experimentally obtained curves and corrected curves) of the Ti-17 alloy with a lamellar (*α*+*β*) starting microstructure tested at different temperatures. (a) (*α*+*β*) region and (b) *β* region. The corrected curves are shown by the squared plots.

Figure 2 shows the stress–strain curves of the (*α*+*β*) region at 750 °C, 800 °C, and 850 °C and the *β* region at 900 °C, 950 °C, and 1050 °C at a strain rates of 10^−3^ s^−1^ and 1 s^−1^ before and after corrections for friction and increasing temperature.

The stresses after friction correction according to the reports [21,22] are calculated when the flow stress and friction factor, *m*_fric_ are substituted into the following equation,
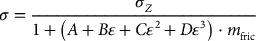



where *σ*_*Z*_ is the true stress obtained experimentally; *m*_fric_ and *ε* are friction factors modified according to the reports [21,22], and true strain, respectively.

The temperature rise due to adiabatic heating during deformation is usually calculated by the following equation [23],
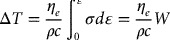



where Δ*T* is the temperature rise due to the work done on the sample, *c* is the heat capacity of material, *ρ* is density of sample, and *W* is the power input into the sample. For example, the estimated Δ*T* is about 53 °C (for testing at 750 °C and 1 s^−1^) and about 0.4 °C (for testing at 750 °C and 10^−3^ s^−1^), respectively. Thus, it can be found that the higher strain rate results in an increase of the deformation heating temperature.

Extensive flow softening is exhibited in the (*α*+*β*) region at higher strain rates. From Figure 2(a), we can observe that the stress values are quite dissimilar before and after correction, especially at higher strain rates. This is mainly due to the effect of adiabatic heating during the deformation at high strain rates. Hence, the continuous flow softening observed at lower temperatures and higher strain rates may be attributed to flow instability, as described elsewhere [2]. Additionally, the lamellae kinking of the *α* phase (as described in the succeeding sections), which frequently occurs at lower temperatures and higher strain rates is also supposed to affect the extensive flow softening, as shown in Figure 2(a). The (*α*+*β*) regions, at conditions of high temperatures and low strain rates (Figure 2(a)), and the *β* regions at high temperatures (Figure 2(b)), exhibited a steady stress behavior, implying that either DRV or CDRX is the dominant mode for microstructural conversion.

#### 3.1.2. Deformed microstructures after forging

**Figure 3.**
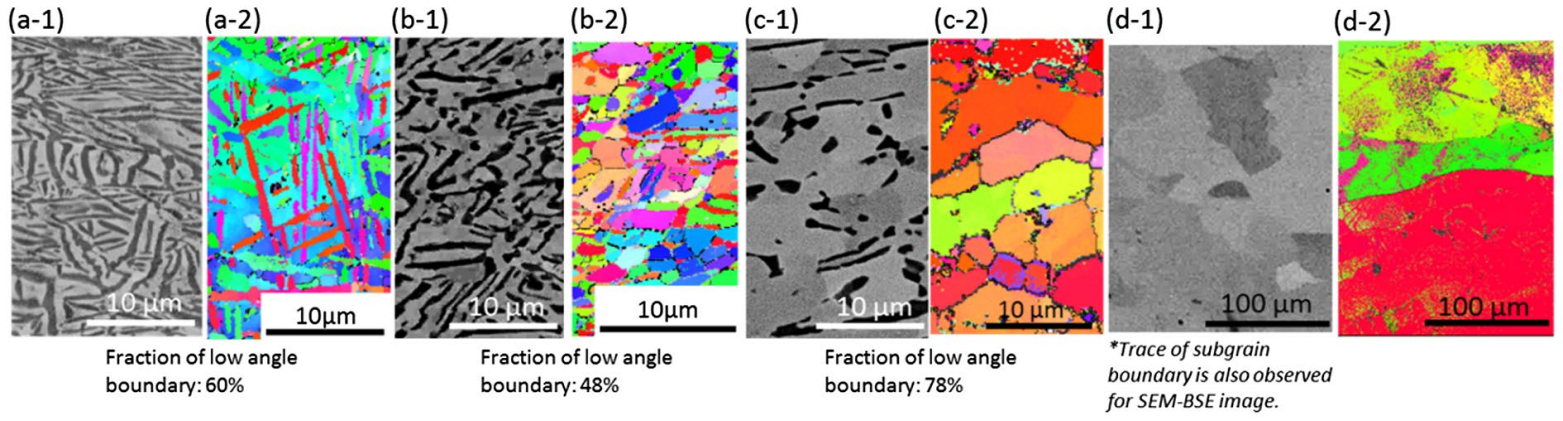
Deformed microstructures of the Ti-17 alloy with a lamellar (*α*+*β*) starting microstructure ((a-1), (b-1), (c-1) and (d-1) depict the SEM-BSE images and (a-2), (b-2), (c-2) and (d-2) depict the EBSD-orientation images) after forging at a strain rate of 10^−3^ s^−1^ and a height true strain of 0.75. The testing temperatures are (a-1) and (a-2) 750 °C, (b-1) and (b-2) 800 °C, (c-1) and (c-2) 850 °C, and (d-1) and (d-2) 950 °C.

Figure 3 summarizes the deformed microstructures (SEM-BSE and EBSD-orientation images) of the Ti-17 alloy after forging at a low strain rate of 10^−3^ s^−1^ and temperatures of 750 °C (Figure 3(a-1) and (a-2)), 800 °C (Figure 3(b-1) and (b-2)), 850 °C (Figure 3(c-1) and (c-2)), and 950 °C (Figure 3(d-1) and (d-2)). The microstructures shown in Figure 3 are observed in the central region of the forged specimens. When the testing temperature was 750 °C, a lamellae kinking phenomenon in which the *α* phase exhibits a bending, resulting in a kinked morphology, could be observed. The decrease in the *α* phase fraction and the frequent activation of dynamic globularization in the *α* phase were found to be dominant with increasing testing temperatures up to 850 °C. The nucleation sites for globularization are at the kinks in the lamellae as well as at some of the prior *β* grain boundaries [24]. This type of microstructural conversion involving a reduction in the aspect ratio of the plates can be interpreted in terms of the geometric dynamic recrystallization proposed by McQueen et al. [8,9]. In the *β* phase, according to the EBSD results shown in Figure 3, the formation of fine equiaxed *β*-grains can be noted at test temperatures of 750 °C and 800 °C. Additionally, it can also be seen from Figure 3 that the fraction of low angle boundary (<15°) (at the *β*/*β* interface) grains decreases in the testing temperature range of 750–800 °C. These results are indicative of the frequent activation of CDRX in the *β* phase in the temperature range of 750–800 °C. On the other hand, coarse *β*-grains with a preferred crystallographic orientation are observed at a test temperature of 850 °C at which the *α* fraction decreases (Figure 3(c-2)). It implies that DRV, instead of CDRX, is enhanced in the *β* phase at 850 °C. This observation is attributable to the decrease in the effect of *α*-pinning on the microstructural conversion in the *β* phase. Consider the deformed microstructure of the *β* region at 1050 °C (Figure 3(d-1) and (d-2)); the SEM-BSE image (Figure 3(d-1)) reveals finer equiaxed *β*-grains with sizes less than the initial grain size of prior *β*-grains (approximately 1 mm). Moreover, the EBSD image (Figure 3(d-2)) reveals that the *β*-grains have preferred crystallographic orientations, which indicates that CDRX is the dominant microstructural conversion mode in the *β* phase.

**Figure 4.**
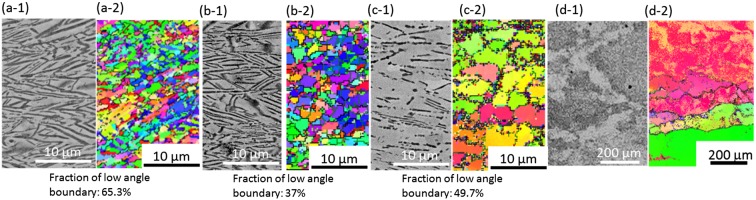
Deformed microstructures of the Ti-17 alloy with a lamellar (*α*+*β*) starting microstructure ((a-1), (b-1), (c-1) and (d-1) depict the SEM-BSE images and (a-2), (b-2), (c-2) and (d-2) depict the EBSD-orientation images) after forging at a strain rate of 10^−1^ s^−1^ and a height true strain of 0.75. The testing temperatures are (a-1) and (a-2) 750 °C, (b-1) and (b-2) 800 °C, (c-1) and (c-2) 850 °C, and (d-1) and (d-2) 950 °C.

The deformed microstructures obtained at a high strain rate of 10^−1^ s^−1^ are summarized in Figure 4. These microstructures were also obtained from the central region in forged specimens. The microstructural conversion mechanism depends on the testing temperature. The characteristics of microstructures obtained seem to be similar to those of the deformed microstructures at lower strain rates (as shown in Figure 3). Compared with the deformed microstructures obtained at a low strain rate of 10^−3^ s^−1^, a smaller fraction of the globularized *α* phase and a finer *β* grain size are noted at a strain rate of 10^−1^ s^−1^. Additionally, in contrast to the results at 10^−3^ s^−1^, it could be observed that the deformed microstructure tested at 850 °C (Figure 4(c-2)) consisted of *β*-grains with a small fraction of low angle boundaries and without an apparent texture formation. This microstructural characteristic reveals that CDRX is more enhanced in the *β* phase at increasing strain rates when the forging temperature was 850 °C. Furthermore, it can be observed that the deformed microstructure tested at 1050 °C (Figure 4(d-1) and (d-2)) consists of a heterogeneous *β* grained microstructure composed of deformed coarse prior *β*-grains and fine equiaxed *β*-grains formed at the boundaries of the coarse *β*-grains.

The apparent activation energy *Q* for hot deformation is determined by assuming that the strain rate follows an Arrhenius type equation, as shown in equation (1)(1)
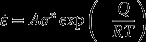
where *A* is the material constant, *R* is the universal gas constant (8.314 J/(mol K)), *n* = (1/*m*) is the stress component, *m* is the strain rate sensitivity defined by *δ*(log *σ*)/*δ*(log 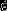), and *T* is the absolute temperature. The estimated *Q* values are 365.4 and 172.8 kJ/mol for the (*α*+*β*) and the *β* regions, respectively. These *Q* values are similar to the *Q* values reported previously for Ti-17 alloys [4]. The higher *Q* value observed for the (*α*+*β*) region is attributable to the occurrence of lamellae kinking, dynamic globularization and DRX. Meanwhile, the *Q* value of the *β* region is similar to the *Q* value (153 kJ/mol) for self-diffusion in a *β*-titanium alloy [25], indicating that the DRV associated with the diffusion process is the dominant mode for forging in the *β* region.

**Figure 5.**
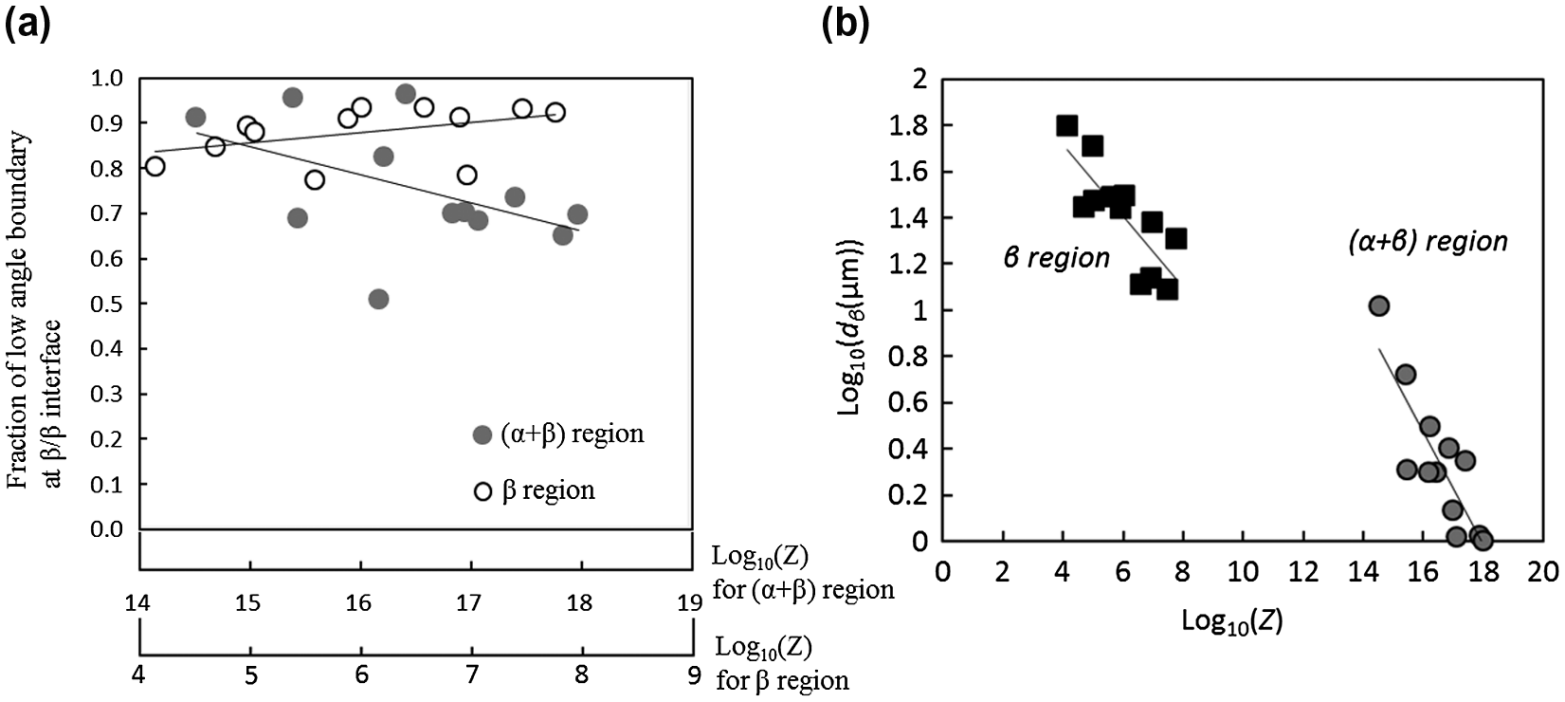
(a) Relationship between *Z* and the fraction of low angle (<15°) boundary (at *β*/*β*) and (b) relationship between *Z* and the average grain size of the *α* phase, *d*_*α*_, in the (*α*+*β*) and *β* region of the forged Ti-17 alloy with a lamellar (*α*+*β*) starting microstructure.

In order to discuss the microstructural conversion mechanism during the deformation of the Ti-17 alloy in more detail, the fractions of low-angle boundaries (at the *β*/*β* interface) in the deformed microstructures in the (*α*+*β*) and *β* regions are summarized as functions of the Zener-Hollomon (*Z*) parameter (in log scale) as shown in Figure 5(a). The *Z* parameter is calculated using Equation (2)(2)
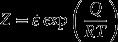



where *R* is the universal gas constant, *T* is the absolute temperature, and *Q* is the activation energy for hot deformation as described above, which indicates that a temperature compensated strain rate parameter is used for kinetic analyzing. There is a strong scattering in the plots obtained by linear approximation, as shown in Figure 5(a). However, we can clearly notice that the fraction of low angle boundaries decreases with an increasing *Z* parameter during deformation in the (*α*+*β*) region; on the other hand, it increases with an increase in the *Z* parameter during deformation in the *β* region. This result indeed points out that CDRX of the *β* phase during deformation in the (*α*+*β*) region and evolution into grains with a large fraction of high angle boundaries are enhanced at testing conditions of low temperatures and high strain rates, whereas DRV is the dominant mode for deformation in the *β* region.

Figure 5(b) shows the relationship between the DRX grain size of the *β* phase and the *Z* parameter in the (*α*+*β*) and *β* regions. Linear relationships can be clearly seen. This result indicates that the grain size itself is dependent on the thermally activated process, which controls the fine grain formation. From Figure 5(b), the relationship between the DRX grain size and the *Z* parameter can be quantitatively summarized as follows.


(3)
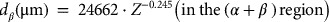



and


(4)
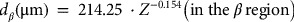



**Figure 6.**
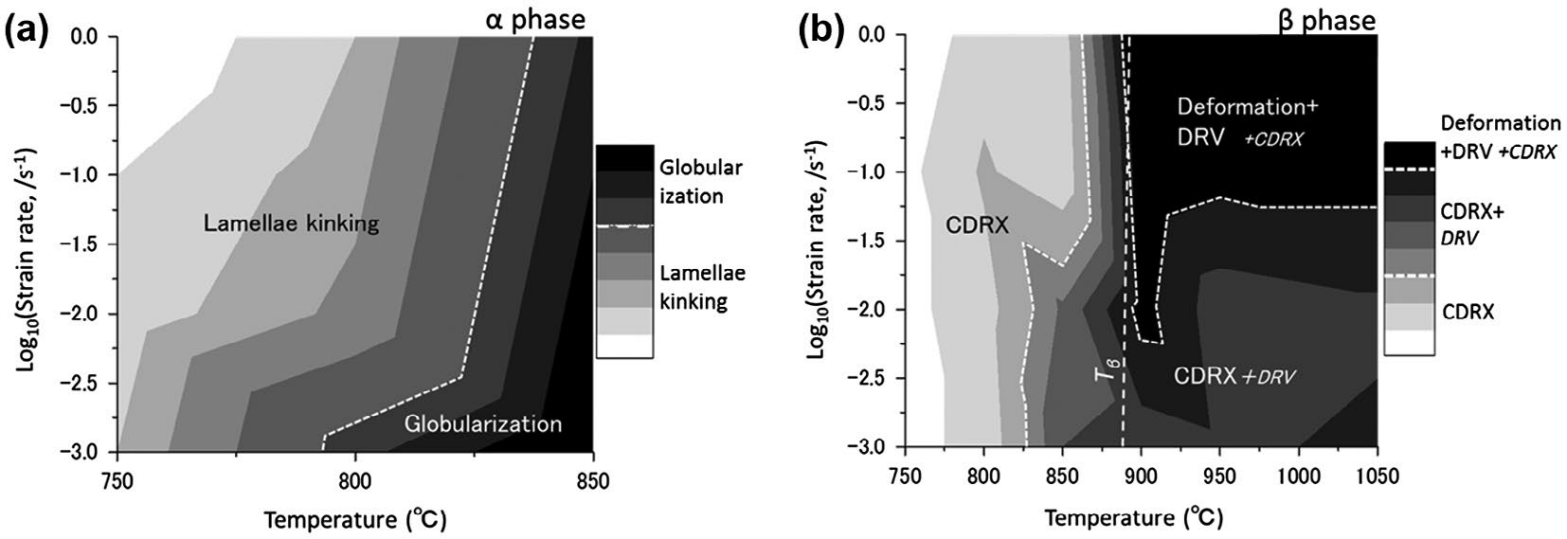
Summary of the microstructural conversion mechanisms as functions of temperature and strain rate after forging in (a) the *α* phase and (b) the *β* phase (indicated by contour map). In the contour map, gradation from white level to black level represents that each respective phenomena of microstructural conversion becomes more dominant.

Figure 6 summarizes the microstructural conversion mechanism in the *α* and *β* phases of the deformed Ti-17 alloy with a lamellar (*α*+*β*) starting microstructure. As described above, lamellae kinking of the *α* phase is the dominant mode in the higher strain rate region and dynamic globularization frequently occurs at high temperatures. The microstructural conversion mechanism in the *β* phase is shown in Figure 6(b), from which it can be understood that CDRX is the dominant mode below *T*_*β*_. DRV tends to be more dominant in conditions of high strain rates and high temperatures. Meanwhile, at temperatures above *T*_*β*_, CDRX frequently occurs, especially in the lower strain rate region.

#### 3.1.3. Effect of differences in the starting microstructure on the forging properties

**Figure 7.**
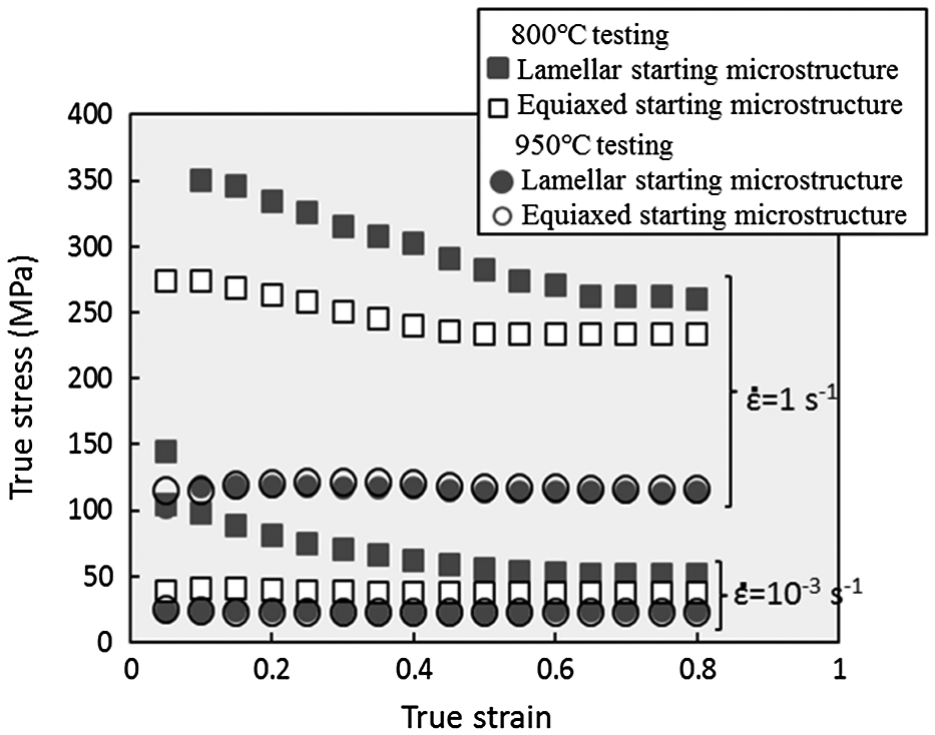
Comparison of the corrected flow curves between the lamellar (*α*+*β*) and the equiaxed (*α*+*β*) starting microstructures for the hot forging of the Ti-17 alloy at 800 °C and 950 °C.

Next, we shall discuss the effect of differences in the starting microstructure on the forging properties, flow behavior and processing map characteristic of the Ti-17 alloy. Figure 7 compares the flow behavior (expressed by corrected stress values as a function of the true strain) of the lamellar (*α*+*β*) starting microstructure and the equiaxed (*α*+*β*) starting microstructure. We can clearly observe the high stress values and extensive flow softening for the lamellar (*α*+*β*) starting microstructure at a testing temperature of 800 °C. According to the results of microstructural conversion described above, this behavior is supposed to occur due to frequent occurrences of lamellae kinking and dynamic globularization in the lamellar (*α*+*β*) starting microstructure. On the other hand, at a testing temperature of 950 °C, similar flow behaviors are exhibited by both microstructures. This implies that the flow behavior of a Ti-17 alloy tested above *T*_*β*_ is independent of the starting microstructure; in contrast, the flow behavior tested below *T*_*β*_ is strongly affected by the *α* phase morphology.

A processing map developed according to the dynamic materials model [18,19] is available for characterizing the forging property of a Ti-17 alloy. This model considers that the workpiece is a power dissipator and that the instant power dissipated at a given 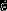 consists of two complementary parts of the *G* content and the *J* content, which are related to the temperature rise and microstructural dissipation, respectively. Processing maps consist of a power dissipation map and an instability map. It enables us to optimize the processing window for the hot working of alloys.

The efficiency of power dissipation, *η*, after normalizing the instantaneous *J* with the maximum value of *J*_max_ can be defined as shown in equation (5).(5)
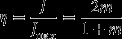



According to Ziegler [26], the continuum criterion for the occurrence of flow instability (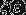) is expressed by equation (3, 6).(6)
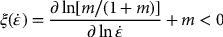



Here, the equation takes the principle of the maximum rate of entropy production into account. Flow instability occurs when 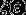 becomes negative; 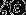 is judged to be metastable when its value lies within the range from –1 to 0. Thus, the processing maps are identified by the variations in *η* and 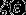 that arise due to variations in the deformation temperature and strain rate.

**Figure 8.**
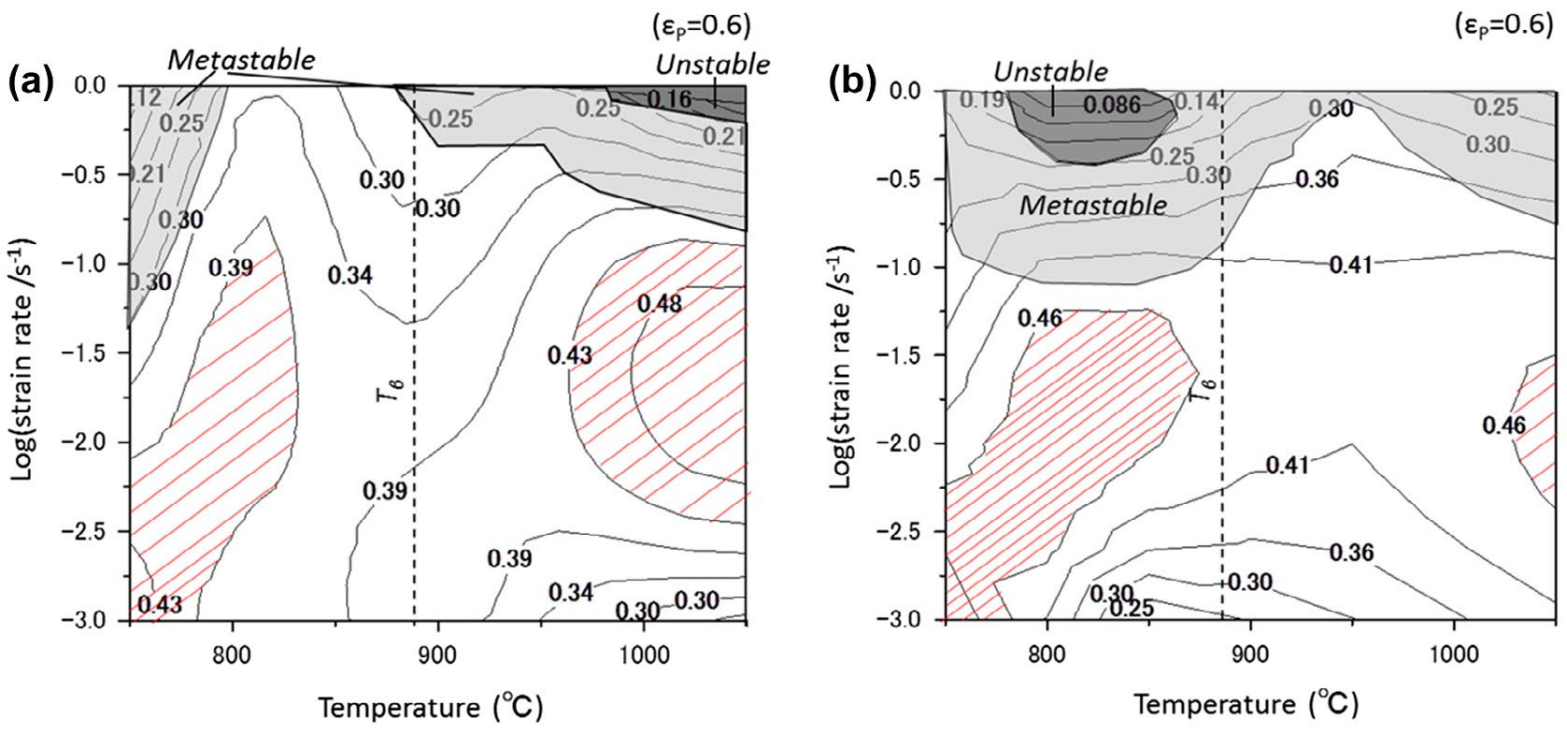
Processing maps (as functions of temperature and strain rate) of isothermally forged Ti-17 alloys in the cases of (a) the lamellar (*α*+*β*) starting microstructure and (b) the equiaxed (*α*+*β*) starting microstructure. The numbers in the contour lines represent the efficiency of power dissipation (*η*) and the shaded area corresponds to the instability region. Additionally, the region with diagonal lines represents the domain exhibiting a high *η* and stable plastic deformation manner, corresponding to an optimum process window for hot working of the alloy.

Figure 8 summarizes the processing maps (at a true strain of 0.6) of the Ti-17 alloy for the lamellar (*α*+*β*) and the equiaxed (*α*+*β*) starting microstructures. The values in the figure represent the efficiency of power dissipation. The shaded areas represent the metastable domains (–1 < 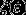 < 0) or the unstable domains (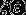 < –1). The figure also points out that a stable deformation is exhibited at strain rates less than 10^−1^ s^−1^ in both of the starting microstructures. The obtained process map in the case of the equiaxed (*α*+*β*) starting microstructure (as shown in Figure 8(b)) is indeed similar to the case in the report on a Ti-17 alloy with an equiaxed starting microstructure [20]. On comparing the processing maps for both starting microstructures, we find that there is no apparent difference in the distribution of *η*. Further, with both the starting microstructures, the metastable- and unstable-domains exist in conditions of high strain rates. The region with the diagonal lines in Figure 8 represents the domains exhibiting higher *η* values and a stable manner, corresponding to the optimum process window for hot working. Herein, we can note that the higher values of *η* in the (*α*+*β*) region are obtained for the equiaxed (*α*+*β*) starting microstructure, implying that a significant microstructural conversion associated with a frequently occurring DRX takes place in the equiaxed (*α*+*β*) starting microstructure. In contrast, at temperatures above *T*_*β*_ in the *β* region, a wide region with a high *η* is found in the lamellar (*α*+*β*) starting microstructure, which is indicative of a frequently occurring CDRX or DRV in the lamellar (*α*+*β*) starting microstructure. To identify the effect of the differences in the starting microstructure on the microstructural conversion mechanism during hot deformation, further experiments are underway along with a detailed study on the deformed microstructures.

### 3.2. Prediction of microstructural evolution and processing map characteristic for forging in the (*α*+*β*) region, along with the FEM analysis

In this work, microstructural prediction focuses on the deformed microstructure in the (*α*+*β*) region. As described above, we can clearly observe microstructural conversion during the dynamic globularization of the *α* phase that is associated with the changes in the aspect ratio and Feret diameter as well as the changes in the grain size of the *β* phase associated with CDRX. Therefore, the constitutive models for these microstructural factors are established as follows.

With respect to dynamic globularization, it is reported that the globularization fraction increases with an increasing strain in a sigmoidal way for the Ti-6Al-4 V alloy [24,27] and the Ti-17 alloy [5]. This behavior is similar to the DRX behavior; therefore, it has been assumed that the dynamic globularization process followed an Avrami type equation [5,28,29]. Similar to earlier reports, the dynamic globularization fraction (*f*_*DG*_) of the *α* phase is defined as follows(7)
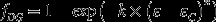



where *f*_*DG*_ is the dynamic globularization fraction, *k* and *n* are material constants, *ε* is the true strain, and *ε*_*C*_ is the critical strain for the onset of dynamic globularization. The *ε*_*C*_ is calculated as(8)
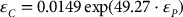



where *ε*_*P*_ corresponds to the true strain at the peak stress in the flow curve (which is experimentally identified) and expressed as follows(9)
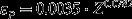



where *Z* is the Zener-Hollomon parameter as expressed in equation (2).

The parameter *k* (in equation (7)), which denotes the reaction rate, is calculated using the following equation, in which the effect of strain rate has been considered.(10)
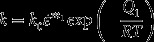



The material constants in the above equations were determined on the basis of the minimization of the sum of errors between the experimental and the calculated data. Herein, the material constants were optimized by non-linear regression analysis. The constants, *k*_0_, *m*_1_, *Q*_1_ and *n*, were determined to be 2895.22, 0.148, 82013 (J/mol) and 2, respectively.

With respect to the modeling of the average aspect ratio and the average Feret-diameter of the *α* phase, constitutive equations were established as expressed in equations (11) and (12), respectively. The material parameters in the equations were established by non-linear regression analysis.(11)
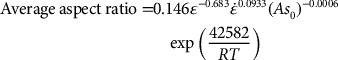

(12)
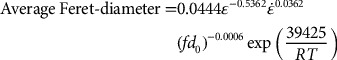



*As*_0_ and *fd*_0_ are initial average aspect ratio of 24 and initial Feret diameter of approximately 4 μm, respectively.

From equations (5) and (6), which evaluate the processing map characteristic, it can be understood that *m* is an important variable for evaluating the processing map. According to the experimental results on the relationship between *m* and log_10_(*Z*) at a true strain of 0.6, the constitutive equation is summarized by an approximated spline-fitting in a cubic polynomial as follows(13)
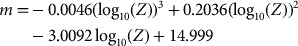



With respect to the efficiency of power dissipation, equation (5) does not consider the effect of strain. For predicting reliable distributions in the processing map characteristic, equation (5) needs to be modified such that it includes the effect of strain on the efficiency of power dissipation. The modified efficiency of power dissipation of *η*′ is hereby defined as follows.(14)
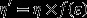




(15)
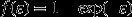



where *ε* is effective strain.

In this equation, the efficiency of the power dissipation is modified by multiplying with the term *f*(*ε*) which is a similar type of equation as the Johnson-Mehl-Avrami-Kolmogorov (JMAK) equation. Thus, the efficiency of power dissipation takes into account the effect of strain, in terms of the *f*(*ε*) parameter.

**Figure 9.**
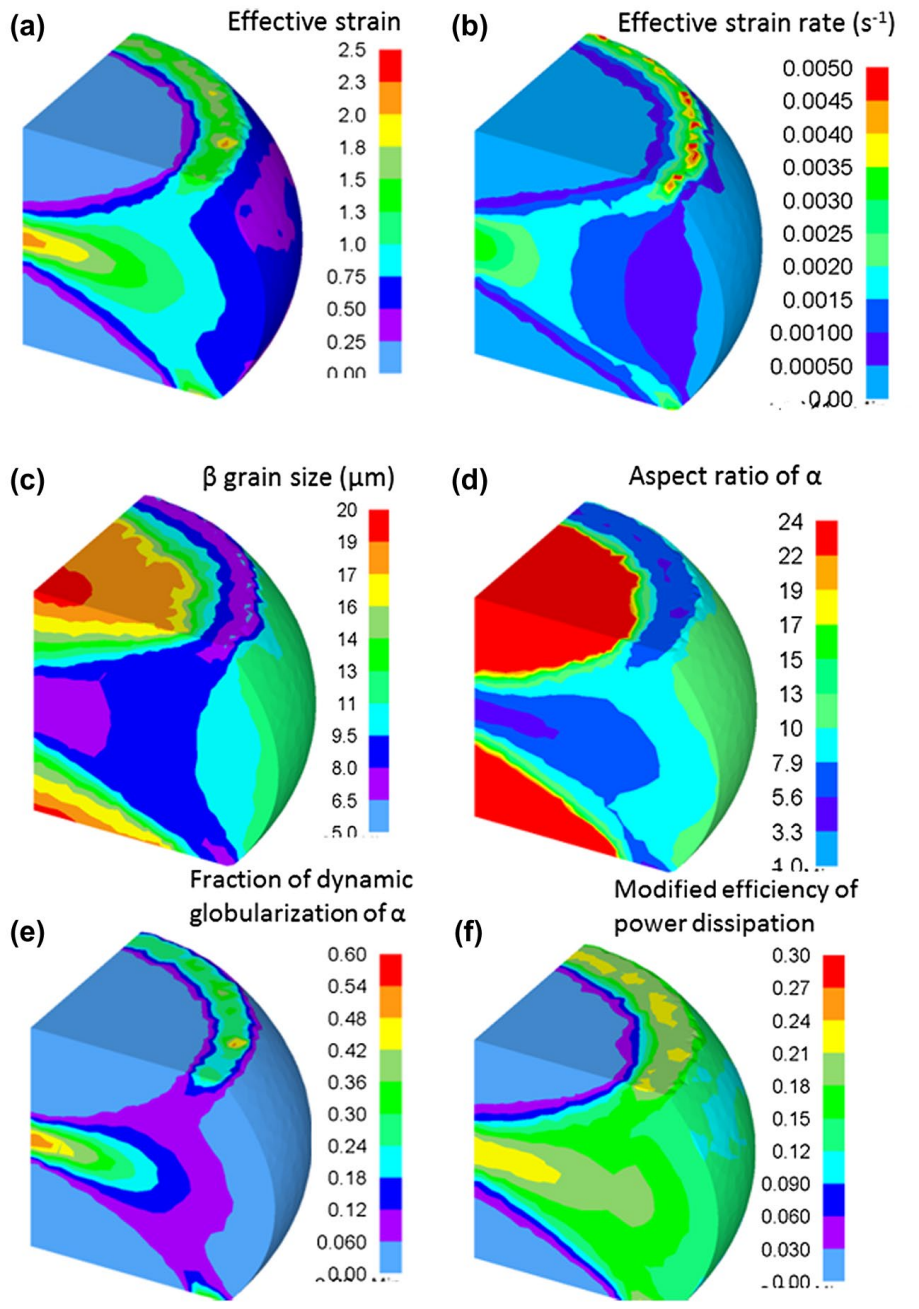
Distributions from the FEM analysis for (a) effective strain, (b) effective strain rate, (c) average *β*-grain size, (d) average aspect ratio of the Feret *α* phase, (e) fraction of dynamic globularization in the *α* phase, and (f) modified efficiency of power dissipation after forging at 850 °C, 10^−3^ s^−1^, and a height true strain of 0.75.

The microstructural evolution model and processing map model (as stated above) were implemented in the DEFORM-3D (ver. 10.2) finite-element software through a user-defined subroutine followed by the simulation of isothermal forging. Figure 9 shows the distributions of effective strain, effective strain rate, *β* grain size, average aspect ratio of the *α* phase, the fraction of dynamic globularization of the *α* phase, and the modified efficiency of power dissipation across the cross-section of the forged specimen tested at 850 °C and 10^−3^ s^−1^. The simulated condition in this study is similar to the experimental condition (as stated in Section 2.1). We can observe from the heterogeneous distribution in Figure 9(a) that strains are locally accumulated, especially in the central region; the effective strain in the central region reaches a maximum value of 2.1. Additionally, there is the low strain region, called the dead zone, in the vicinity of the upper- and lower-end surfaces. Such heterogeneous distributions of the forging property are also observed with respect to the strain rate (as shown in Figure 9(b)) and temperature. In correlation with the distribution of the effective strain (Figure 9(a)), it can be observed from Figure 9(c)–(f) that a finer grain size of the *β* phase, lower aspect ratio of the *α* phase, and a higher fraction of dynamic globularization of the *α* phase result in a higher power dissipation efficiency in the vicinity of the central region of the forged specimen. These results indeed indicate that microstructural conversion during forging occurs mainly in the vicinity of the central region where there is a large accumulation of strains. With respect to the processing map characteristic shown in Figure 9(f), it also exhibits a good correlation with the trends observed in the microstructural conversions of grain size and globularization.

**Figure 10.**
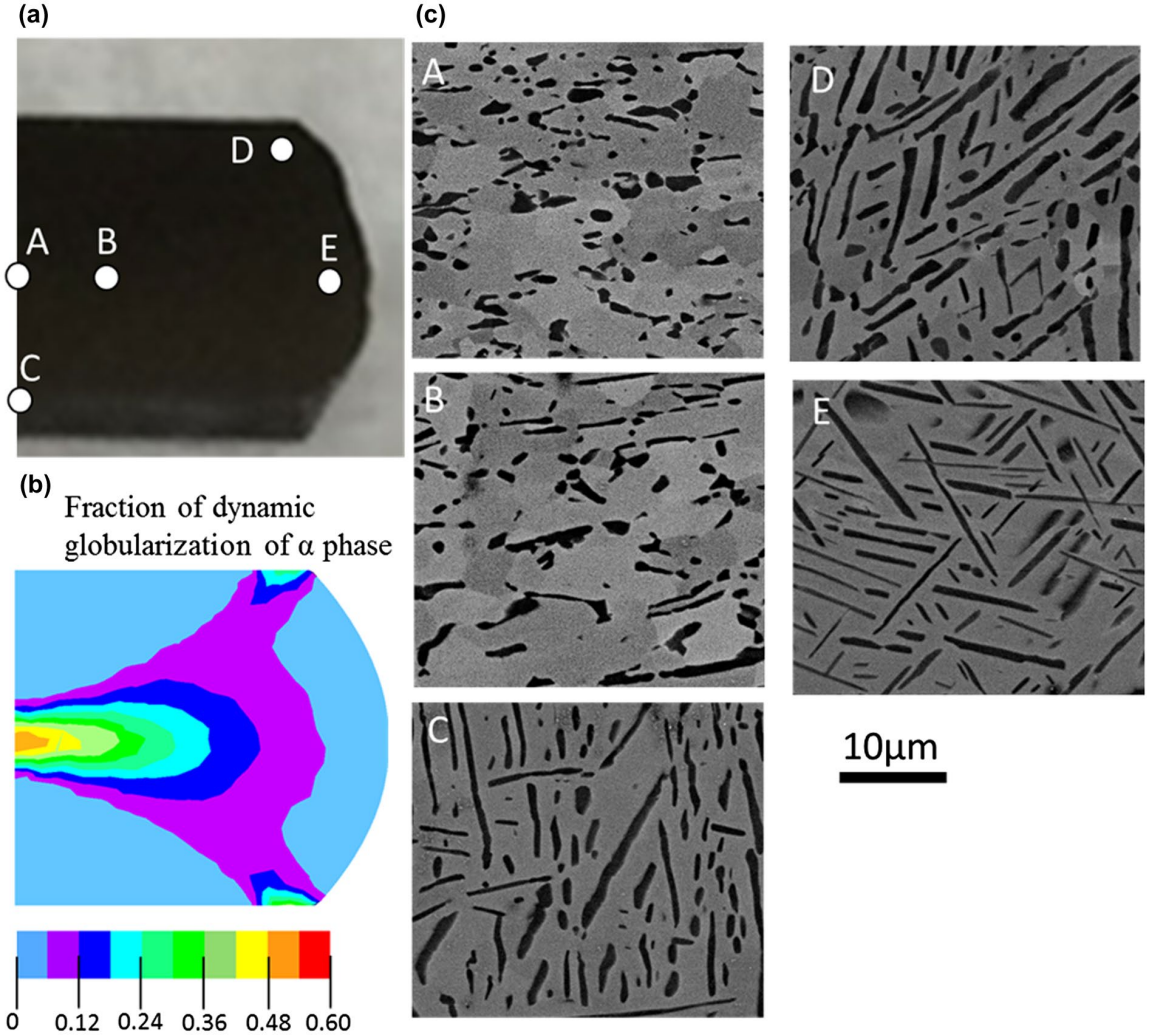
(a) Appearance of the forged Ti-17 specimen at 850 °C, 10^−3^ s^−1^, and a height true strain of 0.75, (b) distribution of dynamic globularization in the *α* phase, as analyzed by FEM, and (c) SEM-BSE images of the forged Ti-17alloy at locations of A–E in (a).

The simulated results are compared with the experimental results (Figure 10). The figure depicts the cross-sectional appearance of the forged specimen, the calculated distribution of the fraction of dynamic globularization of the *α* phase, and the SEM-BSE images at various points (A, B, C, D and E) on the cross-section (as shown in Figure 10(a)). The forging conditions in Figure 10 were 850 °C and 10^−3^ s^−1^. Equiaxed (*α*+*β*) microstructures are obtained at locations A and B. A low fraction of the dynamically globularized *α* phase is observed at D. Additionally, it is observed that the lamellar microstructures are concentrated only at C and E. This reveals that the experimental results are in good agreement with the simulated results. Thus, the constitutive models embedded into the DEFORM-3D platform were found to provide an approximate agreement with the experimental results (microstructures and processing map characteristic).

## 4. Conclusions

We experimentally analyzed the microstructural conversion during the hot forging process of a Ti-17 alloy with a lamellar (*α*+*β*) starting microstructure. In addition, a series of microstructural predictions (dynamic globularization behavior and change in grain size) and modeling of the processing map characteristic for the same alloy were carried out. The results obtained are summarized as follows.

(1) The experimental study on the hot forging of the Ti-17 alloy reveals that lamellae kinking is the dominant microstructural conversion mode in the *α* phase at higher strain rates; dynamic globularization of the *α* phase frequently occurs at high temperatures. On the other hand, CDRX is the dominant mode in the *β* phase at temperatures below *T*_*β*_. Herein, DRV also occurs at lower strain rates. Additionally, deformation associated with the occurrence of DRV is exhibited at testing temperatures above *T*_*β*_ and high strain rates, while CDRX occurred only at prior coarse *β*-grain boundaries. At testing temperatures above *T*_*β*_, the estimated activation energy, *Q*, required for deformation is indeed similar to that required for self-diffusion in the *β* titanium alloy. The processing map characteristics for the lamellar and equiaxed starting microstructures reveal similar behaviors (as functions of temperature and strain rate), which indicates that the hot deformation behavior is not primarily dependent on the starting microstructure.
(2) Constitutive models for microstructural prediction and processing map characteristics were established through an optimization process on the basis of the experimental results, so as to derive the material constants. The dynamic globularization behavior can be reasonably expressed in terms of the modified Avrami type equation. FEM analysis combined with these equations provides an approximate agreement with the experimental results. With respect to the constitutive equations for the processing map, modification using the factor [1–exp(–*ε*)] (which considers the effect of straining) results in a good correlation with the experimental results. Furthermore, the modified equation for efficiency of power dissipation enables the simulation to more reliably correlated with the dynamic globularization behavior. Thus, this work points out the possibility of a reliable prediction of microstructures and processing map characteristics of the Ti-17 alloy after hot forging.

## Disclosure statement

No potential conflict of interest was reported by the authors.

## Funding

This work was supported by the Council for Science, Technology, and Innovation (CSTI), the Cross-ministerial Strategic Innovation Promotion Program (SIP), and the ‘Process Innovation for Super Heat-Resistant Metals (PRISM)’ (Funding agency: JST).
